# DFT Study of the Molecular and Electronic Structure of Metal-Free Tetrabenzoporphyrin and Its Metal Complexes with Zn, Cd, Al, Ga, In

**DOI:** 10.3390/ijms23020939

**Published:** 2022-01-15

**Authors:** Alexey V. Eroshin, Arseniy A. Otlyotov, Ilya A. Kuzmin, Pavel A. Stuzhin, Yuriy A. Zhabanov

**Affiliations:** 1 Research Institute of Chemistry of Macroheterocyclic Compounds, Ivanovo State University of Chemistry and Technology, 153000 Ivanovo, Russia; alexey.yeroshin@gmail.com (A.V.E.); arseniy.otlyotov@chph.ras.ru (A.A.O.); wonderful_37@list.ru (I.A.K.); stuzhin@isuct.ru (P.A.S.); 2 N.N. Semenov Institute of Chemical Physics of Russian Academy of Sciences, Kosygina Street 4, 119991 Moscow, Russia

**Keywords:** tetrabenzoporphyrin, DFT study, molecular and electronic structure, chemical bonding

## Abstract

The electronic and molecular structures of metal-free tetrabenzoporphyrin (**H_2_TBP**) and its complexes with zinc, cadmium, aluminum, gallium and indium were investigated by density functional theory (DFT) calculations with a def2-TZVP basis set. A geometrical structure of **ZnTBP** and **CdTBP** was found to possess D_4h_ symmetry; **AlClTBP**, **GaClTBP** and **InClTBP** were non-planar complexes with C_4v_ symmetry. The molecular structure of **H_2_TBP** belonged to the point symmetry group of D_2h_. According to the results of the natural bond orbital (NBO) analysis, the M-N bonds had a substantial ionic character in the cases of the Zn(II) and Cd(II) complexes, with a noticeably increased covalent contribution for Al(III), Ga(III) and In(III) complexes with an axial –Cl ligand. The lowest excited states were computed with the use of time-dependent density functional theory (TDDFT) calculations. The model electronic absorption spectra indicated a weak influence of the nature of the metal on the Q-band position.

## 1. Introduction

Tetrapyrrole macroheterocycles, such as porphyrines, phthalocyanines and their analogies and metal complexes, have found a number of applications [1,2,3,4]. The possibility of fine-tuning their properties [5] by modification of the peripheral substituents [6] or atoms in the central ring allows us to use them as organic semiconductors, light-emitting diods [7], in photodynamic therapy [8,9], sensors of molecular oxygen [10,11,12] and in medicine, particularly theranostic [13,14,15].

Tetrapyrrolic macrocycles can be used as photosensitizers in photodynamic therapy (PDT), and they often offer imaging capabilities [16,17,18].

The peripheral modification of porphyrin molecules allows the fine-tuning of their spectral luminescence properties, determining their fluorescence and photosensitizing properties and the possibility of attaching peripheral moieties to target penetration in tumor cells [18].

The intense absorption band (Q-band) of tetrabenzoporphyrin generally lies at 600–750 nm, meeting the requirements of an ideal photosensitizer (700–850 nm) [19]. The choice of the range is based on two main factors: the absorption and the scattering of light by tissue decrease as the wavelength increases. Moreover, if the absorption band is too far in the red region, the oxidation potential and photobleaching will be decreased [20,21]. At the same time, the central metal in porphyrins often determines the ratio of the competing fluorescence processes, and internal conversion processes, i.e., efficiency of the singlet oxygen generation. In this regard, along with Zn(II), complex macrocycles containing other heavy metal ions, such as Ga(III) and In(III), are very perspective as photosensitizers. In addition, complexes of phthalocyanines are used as active layers in organic electronic devices, e.g., In(III) phthalocyanine is used as an effective donor in photovolotaic cells [22,23]. The substitution of electronegative meso-nitrogens by meso-CH groups in TPB complexes might be favorable for the enhancement of donor properties.

Complexes of benzo-fused porphyrins, which have more intense absorption in the visible region than common porphyrines, remain much less studied [24,25]; moreover, no information was reported on Ga and In tetrabenzoporphyrins.

Earlier in our laboratory, the properties of Ca(II), Zn(II) Y, La, Lu, metal-free tetra(1,2,5-thiadiazole)porphyrazine [26,27,28], and other analogies of porphyrins, were investigated by quantum chemical calculations.

In this work, we describe the influence of the molecular and electronic structures on the properties of the series of tetrabenzoporphyrin complexes. Quantum chemical calculations were carried out by means of the density functional theory (DFT) [29,30], since our experience shows that it describes macrocyclic metal complexes fairly well [31,32,33,34,35,36,37]. The Ahlrichs’-type def2-basis sets are commonly used for the metal-containing systems [38,39]. At the same time, triple-zeta quality basis sets are normally good enough and less computationally expensive, compared with their quadruple-zeta analogues. Therefore, a def2-TZVP basis set was chosen for the calculations performed in the present work. The nature of the chemical bonding between metal atoms and nitrogen atoms has been described using the NBO-analysis of electron density distribution. The lowest excited states were also calculated in order to explain the peculiarities and tendencies observed in the experimental electronic absorption spectra available for the Zn, Cd and metal-free tetrabenzoporphyrins.

## 2. Results

### 2.1. Molecular Structure

**Figure 1:**
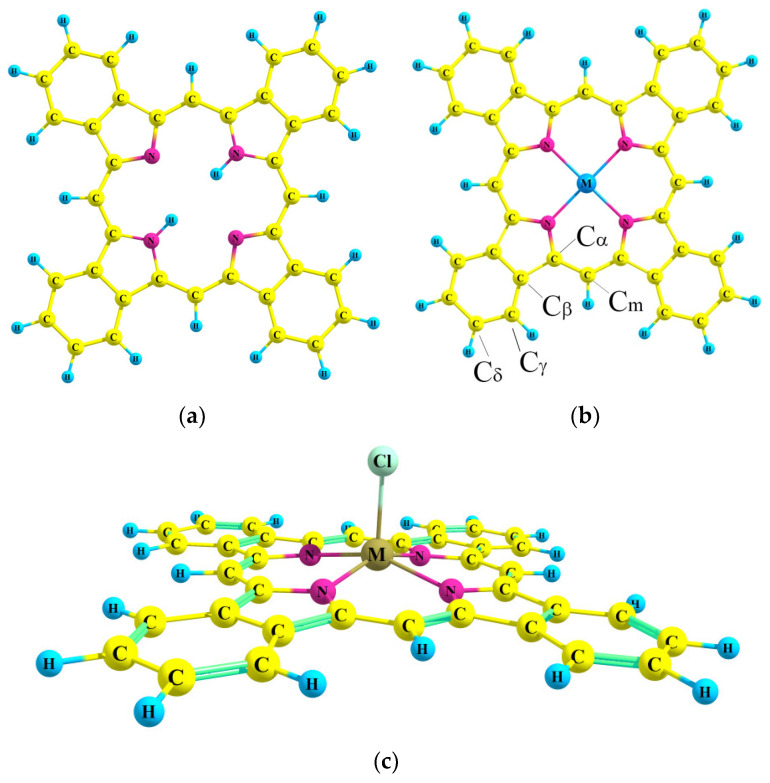
Molecular models of metal-free tetrabenzoporphyrin (**a**), its complexes **MTBP** with Zn, Cd (**b**), **MClTBP** with Al, Ga, In (**c**).

The metal-free tetrabenzoporphyrin molecule **H_2_TBP** possessed a D_2h_ symmetry of equilibrium configuration, according to quantum chemical calculations. All calculated metal complexes had a fourth order symmetry axis: planar (D_4h_) zinc (**ZnTBP**) and cadmium (**CdTBP**) complexes, whereas non-planar with doming-distorted porphyrin skeleton (C_4v_) complexes of aluminum, gallium and indium comprised a chlorine as an axial ligand (**AlClTBP**, **GaClTBP** и **InClTBP**). The optimized molecular structures are depicted in Figure 1.

The degree of the doming distortion for **MClTBP** can be described by the dihedral angle α between planes of opposite pyrrole rings. Its values were 176°, 176° and 172° for **AlClTBP**, **GaClTBP** and **InClTBP**, respectively. The main geometric parameters are listed in Table 1.

The metal out-of-plane distance, defined as the distance between a metal atom and dummy-atom placed in the center of the square of nitrogen atoms, increased from Al to In, which corresponded to an increase in its ionic radii [40]. The influence of the metal nature on the structure of the macrocyclic framework was minor, as in the works [26,27,28]. Indeed, internuclear distances were predominantly intermediate between the corresponding values for the isoindole and isoindolenine fragments of the metal-free tetrabenzoporphyrin. However, the cadmium complex broke out of this trend. There was a significant shortening of the N-C_α_ distance and the C_α_-C_m_ distance elongation) in **CdTBP**.

The M-N distance and C_α_-C_m_-C_α_ valence angle correlated with the ionic radii of metals [40]. The tetrabenzoporphyrines structure resembled both porphyrins and phthalocyanines. It is not surprising that the M-N distance was closer to that in porphyrines [41], rather than in phthalocyanines [42,43], since for the latter the replacement of carbon atoms with nitrogen atoms into meso-positions leads to a decrease in the size of the macrocycle cavity. Our previous studies show that the introduction of peripheral substituents does not significantly affect the structure of the macrocyclic ligand, whereas a change of the metal nature can lead to substantial changes in the inner macrocycle ring internuclear distances.

### 2.2. NBO-Analysis

**Figure 2:**
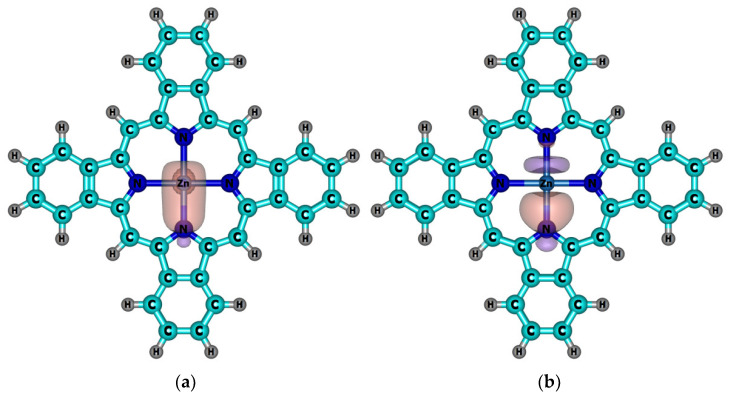
Schemes of the dominant donor–acceptor interactions between Zn and the TBP ligand. (**a**) The result of the orbital interaction of the type LP(N) → 4*s*(Zn) (E(2) = 42.6 kcal mol^−1^); (**b**) the result of the orbital interaction of the type LP(N) → 4*p*(Zn) (E(2) = 35.5 kcal mol^−1^). Only one of the four corresponding interactions is demonstrated.

**Figure 3:**
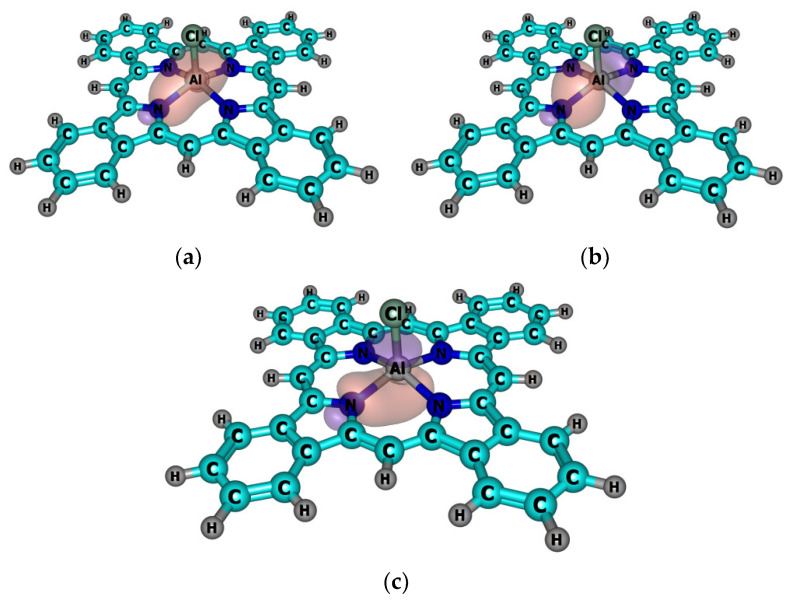
Schemes of the dominant donor–acceptor interactions between the Al and TBP ligand. (**a**) The result of the orbital interaction of the type LP(N) → 3s(Al) (E(2) = 40.7 kcal mol^−1^); (**b**) the result of the orbital interaction of the type LP(N) → 3p_y_(Al) (E(2) = 60.0 kcal mol^−1^); (**c**) the result of the orbital interaction of the type LP(N) → 3p_z_(Al) (E(2) = 12.2 kcal mol^−1^). Only one of the four corresponding interactions is demonstrated.

According to the results of the NBO analysis, the complexes of TBP ligand with Zn(II) and Cd(II) were stabilized by strong interactions of the types: LP(N) → *ns*(M) and LP(N) → *np*(M) (Figure 2; *n* = 4 for Zn, and *n* = 5 for Cd). In the case of the **MClTBP** complexes (M = Al, Ga, In) the out-of-plane position of a metal atom led to an additional favorable overlap LP(N) → *np*_z_(M) (Figure 3, c; *n* = 3 for Al, *n* = 4 for Ga and *n* = 5 for In).

It should be noted that the covalent contribution into the bonds M–N depends on the oxidation state of the central metal, and is higher for M^III^Cl complexes than for complexes of bivalent ions According to the values of the natural charges *q*(M) and the energies of the donor–acceptor interactions (∑ E(d-a)) between lone pairs on the nitrogen atoms and *s*- and *p*-orbitals of the metal atoms, the bond Cd–N was slightly more ionic compared with Zn–N (Table 2). The trend is different for the complexes **MClTBP**: the covalent contribution to the bond M–N increased within the series Al → Ga → In. The enhanced covalent properties of Ga–N bond correlated with the well-known [44,45] alternation in the electronegativities of Al (χ = 1.47), Ga (χ = 1.82) and In (χ = 1.49).

### 2.3. Electronic Spectra

**Figure 4:**
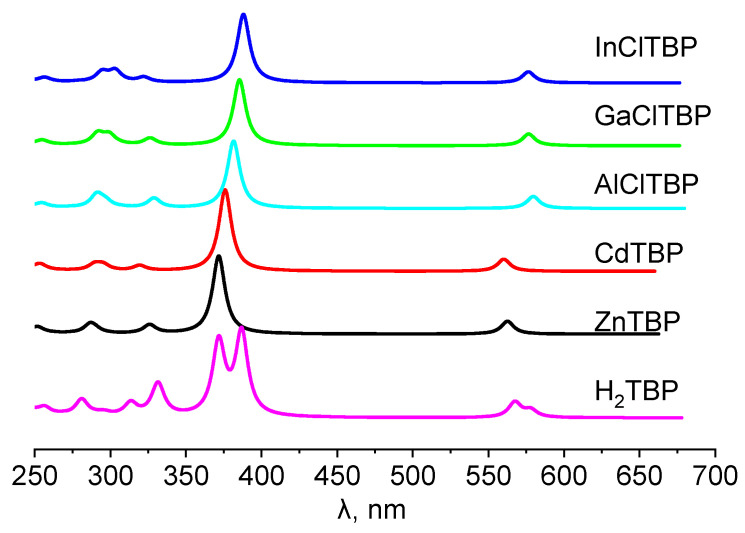
Calculated TDDFT electronic absorption spectra for **H_2_TBP**, **MTBP** and **MClTBP** complexes.

The calculated spectra of the investigated compounds were quite similar, which demonstrates the insignificant influence of the metal nature to the position of the Q-band. In the spectrum of **H_2_TBP**, the splitting of the Q-band into two bands was observed. Q-band splitting occurred because of the double-degenerated LUMOs of e_g_* symmetry of **MTBP,** which split into two *b*_1g_* and *b*_2g_* orbitals of **H_2_TBP**, since the latter had a lower symmetry compared with the metal complexes. A negligible batochromic shift for the complexes with axial ligands, and a hypsochromic shift for Cd and Zn occurred with a metal being introduced into the **H_2_TBP** molecule (Figure 4).

The calculated oscillator strengths (f) for the lowest-allowed excited states, along with their composition (in terms of one-electron transition) are given in Table 3. A full version of the table is listed in Table S1.

The long-wave absorption maxima (Q band) in the spectra of **MTBP** can be assigned to the transitions from HOMO and HOMO-1 to the double-degenerated LUMO. This is typical for macroheterocycles such as porphyrazines with Ca, Zn [27] and -tetrakis(1,2,5-thiadiazole)porphyzarines with Y, La, Lu [28]. The shift of the long-wave maximum towards higher values in the case of MClTBP, compared with MTBP, was quite small (*ca*. 20 nm) and might be attributed either to the different nature of a central metal atom, or to the presence of the axial chlorine substituent. The most intensive peak in the spectra, the Soret band, predominantly corresponded to the electron transitions from the occupied *a*_1_ (Al, Ga, In complexes) *a*_2*u*_ (Zn, Cd complexes)-type MOs to the LUMOs. The composition of the Q- and Soret-bands were quite similar for all complexes, and can be described by the model of Gouterman [46,47].

**Figure 5:**
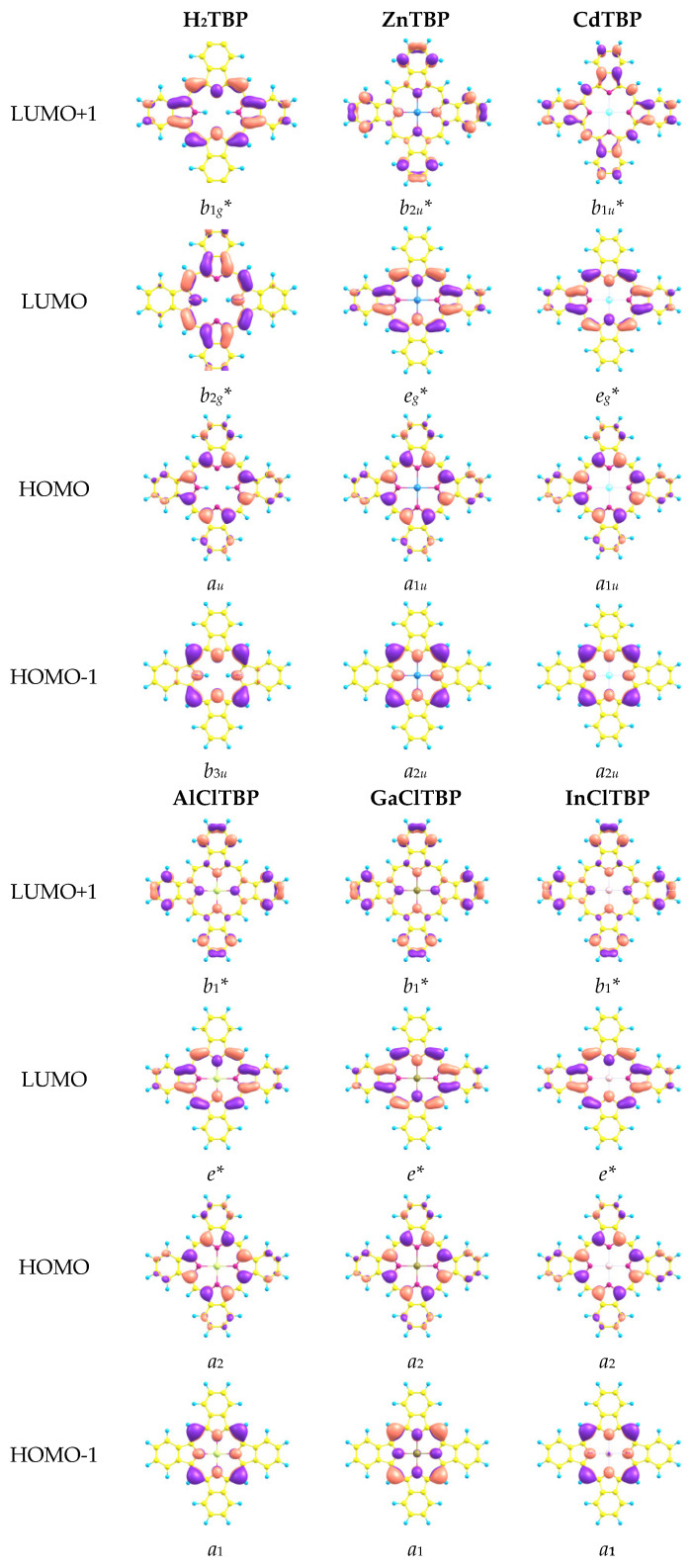
Shapes of the frontier molecular orbitals.

Shapes of the highest occupied molecular orbitals (HOMO), HOMO-1 and lowest unoccupied molecular orbitals (LUMO) were similar (Figure 5) for all the investigated complexes. HOMO is an *a_u_* (H_2_), *a*_1*u*_ (Zn, Cd) or *a*_2_ (Al, Ga, In), LUMOs are doubly-degenerated e* or e_g_* orbitals. The symmetry of these orbitals is typical for porphyrines and porphyrazines [27,31,51,52].

The HOMO predominantly represented the linear combination of atomic orbitals (AOs) of the pyrrole rings, whereas the HOMO-1 was localized on the carbon atoms in the meso-positions. Furthermore, HOMO-1 and HOMO-2 orbitals contained both AOs of the macrocycle and axial ligand Cl for Al, Ga and In complexes. The contribution of the metal to this orbital was slight.

**Figure 6:**
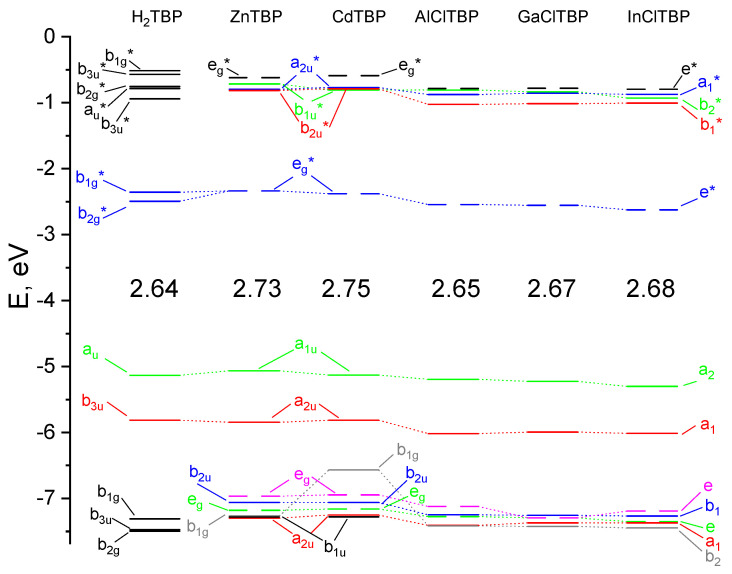
Molecular orbital (MO) level diagram for H_2_TBP and MTBP complexes. The values of higher occupied molecular orbital–lowest unoccupied molecular orbital (HOMO–LUMO) gaps are given in eV.

Despite the fact that the HOMO–LUMO gap was the lowest for metal-free **H_2_TBP** (Figure 6), the wavelength of its Q-band was not the largest among the molecules considering both the transitions from HOMO to LUMO, and from HOMO-1 to LUMO, contributed to Q-band, according to the quantum chemical calculations performed.

## 3. Computational Details

Quantum chemical calculations of the tetrabenzoporphyrin and its metal complexes with Zn, Cd, Al, Ga, and In were performed using the Gaussian09 [53] program. PBE0 exchange-correlation (XC) functional with the density functional dispersion correction D3 provided by Grimme [54], in combination with the def2-TZVP basis set [55] for all atoms taken from the EMSL BSE library [56,57,58], was applied for the structure optimization and computation of harmonic vibrations. Analytic Hessian calculations indicated the absence of the imaginary vibrational frequencies and, therefore, the optimized structures corresponded to the minima on the PES. The optimized Cartesian coordinates of **H_2_TBP** and its metal complexes with Zn, Cd, Al, Ga, and In are available in the Supplementary Materials.

For describing the core electron shells of the cadmium and indium atoms, pseudopotentials combined with a corresponding basis set were used. The doubly occupied orbitals corresponding to the 1s, 2s, 2p, 3s, 3p and 3d orbitals were described by multiconfiguration-Dirac–Hartree–Fock-adjusted pseudopotentials [59,60].

Gaussian03 [61] was employed for the NBO-analysis of electron density distribution. TDDFT calculations of the electronic absorption spectra were performed with the use of the Firefly QC package [62], which is partially based on the GAMESS (US) [63] source code, since it supports separate computations of the electronic transitions for each irreducible representation which, in turn, results in the proper automatic determination of the wave function symmetry. The number of the calculated excited states was 30.

The molecular models and orbitals demonstrated in the paper were visualized by means of the Chemcraft program [64].

## 4. Conclusions

The influence of the nature of the metal (M = Zn, Cd, Al(Cl), Ga(Cl), In(Cl)) on the molecular and electronic structure of the tetrabenzoporphyrin molecule **H_2_TBP** was studied using the DFT method (PBE0 functional) with a def2-TZVP basis set. A weak influence of the metal nature on the structure of the macrocyclic framework was observed, whereas the dimensions of the coordination cavity of the macrocycle increased in proportion to the ionic radii of metals. The electron density distribution was considered in terms of the natural bond orbitals (NBO). The low values of the Wyberg bond indices indicated that the M-N bonds had an ionic character with a noticeable covalent contribution, which depended on the oxidation state of the metal. The complexes were stabilized by strong interactions of the types: LP(N) → *ns*(M), LP(N) → *np*(M). For non-planar complexes with **Al**, **Ga**, and **In**, an additional favorable overlap LP(N) → *np*_z_(M) appeared, where n was a principal quantum number.

The Q-band position was weakly dependent on the nature of the metal. The frontier orbitals were mainly localized on the atoms constituting the internal 16-membered macrocycle. HOMO and LUMOs were found to be ordinary Gouterman-type orbitals. The HOMO–LUMO gap was the lowest for metal-free **H_2_TBP**; however, an insignificant batochromic shift of the Q-band for the complexes with axial ligand, and a hypsochromic shift of the Q-band for Cd and Zn occurred, compared with the metal-free **H_2_TBP** molecule, since both the transitions from HOMO to LUMO and from HOMO-1 to LUMO contributed to the Q-band.

## Figures and Tables

**Table 1:** Molecular parameters ^1^ of H_2_-tetrabenzoporphyrin and its metal complexes optimized at PBE0/def2-TZVP level.

	H_2_TBP	ZnTBP	CdTBP	AlClTBP	GaClTBP	InClTBP
Symmetry	*D*_2h_	*D*_4h_	*D*_4h_	*C*_4v_	*C*_4v_	*C*_4v_
Distances						
M-N	1.012 (2.340) ^2^	2.063	2.152	2.044	2.075	2.184
M-Cl	-	-	-	2.154	2.196	2.360
N…N_opp_	4.268 (4.106)	4.125	4.304	4.006	4.060	4.195
N…N_adj_	2.961	2.917	3.043	2.833	2.871	2.966
N-C_α_	1.362 (1.353)	1.363	1.355	1.370	1.366	1.361
C_α_-C_β_	1.439 (1.457)	1.446	1.453	1.440	1.441	1.446
C_β_-C_β_	1.407 (1.399)	1.401	1.409	1.395	1.397	1.404
C_β_-Cγ	1.394 (1.389)	1.393	1.391	1.394	1.393	1.392
Cγ-C_δ_	1.379 (1.385)	1.381	1.383	1.380	1.380	1.381
C_δ_-C_δ_	1.404 (1.398)	1.402	1.399	1.403	1.402	1.401
C_α_-C_m_	1.379 (1.390)	1.383	1.398	1.373	1.376	1.387
C_α_-C_m_-C_α_	128.0	127.4	130.3	125.3	126.0	128.3
r(M-X) ^3^	-	0	0	0.407	0.453	0.678
Bond angles						
N-C_α_-C_m_	126.2 (125.9)	125.6	125.6	125.6	125.8	125.8
N-C_α_-C_β_	106.3 (110.7)	109.5	107.6	110.6	109.9	108.6
A ^4^	180.0	180.0	180.0	176.3	176.4	171.6

^1^ All internuclear distances are in Angstroms (Å), valence angle are in degrees (°). ^2^ The values in parentheses correspond to the corresponding values for the isoindolenine fragments of the metal-free tetrabenzoporphyrin. ^3^ X is a dummy atom located in the center between N atoms. ^4^ α is the dihedral angle between planes of opposite pyrrole rings.

**Table 2:** Selected parameters of MTBP complexes from NBO calculations.

	ZnTBP	CdTBP	AlClTBP	GaClTBP	InClTBP
*E*(HOMO), eV	−5.06	−5.13	−5.19	−5.22	−5.30
*E*(LUMO), eV	−2.34	−2.38	−2.54	−2.55	−2.62
∆*E*, eV	2.72	2.75	2.65	2.67	2.68
*q*(M) NPA, *e*	1.304	1.333	1.776	1.673	1.718
*q*(N) NPA, *e*	−0.573	−0.570	−0.607	−0.584	−0.579
*q*(Cl) NPA, *e*			−0.574	−0.545	−0.563
configuration	4s^0.35^3d^9.97^4p^0.37^	5s^0.41^5d^9.95^5p^0.31^	3s^0.42^3p^0.76^	4s^0.55^4p^0.76^	5s^0.54^5p^0.73^
∑ *E*(d-a), kcal mol^−1^	312.2	303.8	451.7	495.6	471.9
*Q*(M–N)	0.283	0.274	0.320	0.335	0.330
*r*(M–N), Å	2.063	2.152	2.044	2.075	2.184

**Table 3:** Calculated composition of the lowest excited states and corresponding oscillator strengths for H_2_TBP and MTBP complexes.

State	Composition (%)	λ, nm	f	exp λ, nm
**H_2_TBP**				
1^1^B_1u_	$2b_{3u}\to 1b_{2g}^{*}\;$(31) $3a_{u}\to 1b_{1g}^{*}$ (69)	578	0.11	663.5 (Py) [48]
1^1^B_2u_	$2b_{3u}\to 1b_{1g}^{*}\;$(19) $3a_{u}\to 1b_{2g}^{*}\;$(81)	568	0.23	
2^1^B_1u_	$2b_{3u}\to 1b_{2g}^{*}\;$(8) $2b_{3u}\to 1b_{2g}^{*}\;$(59) $3a_{u}\to 1b_{1g}^{*}$ (28)	387	1.24	431.8 (Py) [48]
2^1^B_2u_	$2b_{3u}\to 1b_{1g}^{*}\;$(74) $3a_{u}\to 1b_{2g}^{*}\;$(18)	372	1.10	416.1 (Py) [48]
3^1^B_1u_	$2b_{3u}\to 1b_{2g}^{*}\;$(88) $2b_{3u}\to 1b_{2g}^{*}\;$(6)	332	0.35	
3^1^B_2u_	$3a_{u}\to 2b_{2g}^{*}\;$(93)	331	0.15	
**ZnTBP**				
1^1^ E_u_	$2a_{2u}\to 1e_{g}^{*}\;$(21) $2a_{1u}\to 1e_{g}^{*}$ (79)	563	0.19	613(Ar matrix) [49] 628.5 (Py) [48]
2^1^ E_u_	$2a_{2u}\to 1e_{g}^{*}\;$(73) $2a_{1u}\to 1e_{g}^{*}$ (20)	372	1.17	433.3(Py) [48]
3^1^ E_u_	$2a_{1u}\to 2e_{g}^{*}$ (94)	326	0.13	
**CdTBP**				
1^1^E_u_	$2a_{2u}\to 1e_{g}^{*}\;$(23) $2a_{1u}\to 1e_{g}^{*}$ (77)	560	0.18	628 (Py) [50]
2^1^ E_u_	$2a_{2u}\to 1e_{g}^{*}\;$(71) $2a_{1u}\to 1e_{g}^{*}$ (22)	376	1.21	434 (Py) [50]
3^1^E_u_	$1b_{2u}\to 1e_{g}^{*}$ (18) $2a_{1u}\to 2e_{g}^{*}$ (78)	320	0.08	
**AlClTBP**				
1^1^E	$2a_{1}\to 1e^{*}\;$(20) $2a_{2}\to 1e^{*}$ (80)	580	0.18	
2^1^E	$2a_{1}\to 1e^{*}\;$(73) $2a_{2}\to 1e^{*}$ (19)	382	1.01	
3^1^E	$2a_{2}\to 2e^{*}$ (93)	329	0.14	
**GaClTBP**				
1^1^E	$3a_{1}\to 1e^{*}\;$(21) $2a_{2}\to 1e^{*}$ (79)	577	0.17	
2^1^E	$3a_{1}\to 1e^{*}\;$(73) $2a_{2}\to 1e^{*}$ (20)	385	0.99	
3^1^E	$2b_{1}\to 1e^{*}\;$(5) $2a_{2}\to 2e^{*}$ (93)	326	0.10	
**InClTBP**				
1^1^E	$2a_{1}\to 1e^{*}\;$(22) $2a_{2}\to 1e^{*}$ (77)	577	0.17	
2^1^E	$2a_{1}\to 1e^{*}\;$(72) $2a_{2}\to 1e^{*}$ (22)	388	1.03	
3^1^E	$1b_{1}\to 1e^{*}\;$(23) $2a_{2}\to 2e^{*}$ (73)	322	0.07	

## Data Availability

The data presented in this study are available on request from the corresponding author.

## Supplementary Materials

The following supporting information can be downloaded at: https://www.mdpi.com/article/10.3390/ijms23020939/s1.
