# Supplementary material for: DFT Study of the Molecular and Electronic Structure of Metal-Free Tetrabenzoporphyrin and Its Metal Complexes with Zn, Cd, Al, Ga, In
Source: Int J Mol Sci. 2022 Jan 15;23(2):939. doi: 10.3390/ijms23020939 (PMC8781462; doi:10.3390/ijms23020939)
Supplement: Supplementary file 1 [file ijms-23-00939-s001.zip › ijms-1552472-supplementary.pdf]

Supporting information for

«DFT Study of Molecular and Electronic Structure of Metal-free Tetrabenzoporphyrin and its Metal Complexes with Zn, Cd, Al, Ga, In»

Alexey V. Eroshin, Arseniy A. Otlyotov, Ilya A. Kuzmin, Pavel A. Stuzhin and Yuriy A. Zhabanov

Content

Cartesian coordinates of H_2TBP optimized PBE0/def2-TZVP level of theory: 2

Cartesian coordinates of ZnTBP optimized PBE0/def2-TZVP level of theory: 3

Cartesian coordinates of CdTBP optimized PBE0/def2-TZVP level of theory: 4

Cartesian coordinates of AlClTBP optimized PBE0/def2-TZVP level of theory: 5

Cartesian coordinates of GaClTBP optimized PBE0/def2-TZVP level of theory: 6

Cartesian coordinates of InClTBP optimized PBE0/def2-TZVP level of theory: 8

Table S1. Calculated composition of the lowest excited states and corresponding oscillator strengths for H_2TBP and MTBP complexes....... 10

Cartesian coordinates of H_2TBP optimized PBE0/def2-TZVP level of theory:

H	0.000000000000	-1.231106000000	7.588808000000
N	0.000000000000	2.053087000000	0.000000000000
N	0.000000000000	0.000000000000	-2.134022000000
N	0.000000000000	-2.053087000000	0.000000000000
N	0.000000000000	0.000000000000	2.134022000000
C	0.000000000000	2.428386000000	-2.413860000000
C	0.000000000000	-2.428386000000	-2.413860000000
C	0.000000000000	-2.428386000000	2.413860000000
C	0.000000000000	2.428386000000	2.413860000000
C	0.000000000000	2.853751000000	-1.090613000000
C	0.000000000000	-1.133675000000	-2.889127000000
C	0.000000000000	-2.853751000000	1.090613000000
C	0.000000000000	1.133675000000	2.889127000000
C	0.000000000000	2.853751000000	1.090613000000
C	0.000000000000	1.133675000000	-2.889127000000
C	0.000000000000	-2.853751000000	-1.090613000000
C	0.000000000000	-1.133675000000	2.889127000000
C	0.000000000000	4.257078000000	-0.699292000000
C	0.000000000000	-0.703654000000	-4.262556000000
C	0.000000000000	-4.257078000000	0.699292000000
C	0.000000000000	0.703654000000	4.262556000000
C	0.000000000000	4.257078000000	0.699292000000
C	0.000000000000	0.703654000000	-4.262556000000
C	0.000000000000	-4.257078000000	-0.699292000000
C	0.000000000000	-0.703654000000	4.262556000000
C	0.000000000000	5.449843000000	-1.411178000000
C	0.000000000000	-1.413755000000	-5.462103000000
C	0.000000000000	-5.449843000000	1.411178000000
C	0.000000000000	1.413755000000	5.462103000000
C	0.000000000000	5.449843000000	1.411178000000
C	0.000000000000	1.413755000000	-5.462103000000
C	0.000000000000	-5.449843000000	-1.411178000000
C	0.000000000000	-1.413755000000	5.462103000000
C	0.000000000000	6.637578000000	-0.698990000000
C	0.000000000000	-0.701771000000	-6.642976000000
C	0.000000000000	-6.637578000000	0.698990000000
C	0.000000000000	0.701771000000	6.642976000000
C	0.000000000000	6.637578000000	0.698990000000
C	0.000000000000	0.701771000000	-6.642976000000
C	0.000000000000	-6.637578000000	-0.698990000000
C	0.000000000000	-0.701771000000	6.642976000000
H	0.000000000000	5.461641000000	-2.495644000000
H	0.000000000000	-2.497666000000	-5.471653000000
H	0.000000000000	-5.461641000000	2.495644000000
H	0.000000000000	2.497666000000	5.471653000000
H	0.000000000000	5.461641000000	2.495644000000
H	0.000000000000	2.497666000000	-5.471653000000
H	0.000000000000	-5.461641000000	-2.495644000000
H	0.000000000000	-2.497666000000	5.471653000000
H	0.000000000000	7.582863000000	-1.229777000000
H	0.000000000000	7.582863000000	1.229777000000

H	0.000000000000	-7.582863000000	-1.229777000000
H	0.000000000000	-7.582863000000	1.229777000000
H	0.000000000000	-1.231106000000	-7.588808000000
H	0.000000000000	1.231106000000	-7.588808000000
H	0.000000000000	1.231106000000	7.588808000000
H	0.000000000000	0.000000000000	1.121758000000
H	0.000000000000	0.000000000000	-1.121758000000
H	0.000000000000	-3.203021000000	3.171414000000
H	0.000000000000	-3.203021000000	-3.171414000000
H	0.000000000000	3.203021000000	3.171414000000
H	0.000000000000	3.203021000000	-3.171414000000

Cartesian coordinates of ZnTBP optimized PBE0/def2-TZVP level of theory:

H	7.573609000000	-1.230468000000	0.000000000000
N	0.000000000000	2.062632000000	0.000000000000
N	-2.062632000000	0.000000000000	0.000000000000
N	0.000000000000	-2.062632000000	0.000000000000
N	2.062632000000	0.000000000000	0.000000000000
C	-2.416080000000	2.416080000000	0.000000000000
C	-2.416080000000	-2.416080000000	0.000000000000
C	2.416080000000	-2.416080000000	0.000000000000
C	2.416080000000	2.416080000000	0.000000000000
C	-1.106164000000	2.859050000000	0.000000000000
C	-2.859050000000	-1.106164000000	0.000000000000
C	1.106164000000	-2.859050000000	0.000000000000
C	2.859050000000	1.106164000000	0.000000000000
C	1.106164000000	2.859050000000	0.000000000000
C	-2.859050000000	1.106164000000	0.000000000000
C	-1.106164000000	-2.859050000000	0.000000000000
C	2.859050000000	-1.106164000000	0.000000000000
C	-0.700532000000	4.247120000000	0.000000000000
C	-4.247120000000	-0.700532000000	0.000000000000
C	0.700532000000	-4.247120000000	0.000000000000
C	4.247120000000	0.700532000000	0.000000000000
C	0.700532000000	4.247120000000	0.000000000000
C	-4.247120000000	0.700532000000	0.000000000000
C	-0.700532000000	-4.247120000000	0.000000000000
C	4.247120000000	-0.700532000000	0.000000000000
C	-1.412775000000	5.443812000000	0.000000000000
C	-5.443812000000	-1.412775000000	0.000000000000
C	1.412775000000	-5.443812000000	0.000000000000
C	5.443812000000	1.412775000000	0.000000000000
C	1.412775000000	5.443812000000	0.000000000000
C	-5.443812000000	1.412775000000	0.000000000000
C	-1.412775000000	-5.443812000000	0.000000000000
C	5.443812000000	-1.412775000000	0.000000000000
C	-0.700884000000	6.627663000000	0.000000000000
C	-6.627663000000	-0.700884000000	0.000000000000
C	0.700884000000	-6.627663000000	0.000000000000
C	6.627663000000	0.700884000000	0.000000000000
C	0.700884000000	6.627663000000	0.000000000000
C	-6.627663000000	0.700884000000	0.000000000000

C	-0.700884000000	-6.627663000000	0.000000000000
C	6.627663000000	-0.700884000000	0.000000000000
H	-2.496991000000	5.454885000000	0.000000000000
H	-5.454885000000	-2.496991000000	0.000000000000
H	2.496991000000	-5.454885000000	0.000000000000
H	5.454885000000	2.496991000000	0.000000000000
H	2.496991000000	5.454885000000	0.000000000000
H	-5.454885000000	2.496991000000	0.000000000000
H	-2.496991000000	-5.454885000000	0.000000000000
H	5.454885000000	-2.496991000000	0.000000000000
H	-1.230468000000	7.573609000000	0.000000000000
H	1.230468000000	7.573609000000	0.000000000000
H	-1.230468000000	-7.573609000000	0.000000000000
H	1.230468000000	-7.573609000000	0.000000000000
H	-7.573609000000	-1.230468000000	0.000000000000
H	-7.573609000000	1.230468000000	0.000000000000
H	7.573609000000	1.230468000000	0.000000000000
H	3.182285000000	-3.182285000000	0.000000000000
H	-3.182285000000	-3.182285000000	0.000000000000
H	3.182285000000	3.182285000000	0.000000000000
H	-3.182285000000	3.182285000000	0.000000000000
Zn	0.000000000000	0.000000000000	0.000000000000

Cartesian coordinates of CdTBP optimized PBE0/def2-TZVP level of theory:

H	7.635111000000	-1.230654000000	0.000000000000
N	0.000000000000	2.152072000000	0.000000000000
N	-2.152072000000	0.000000000000	0.000000000000
N	0.000000000000	-2.152072000000	0.000000000000
N	2.152072000000	0.000000000000	0.000000000000
C	-2.433355000000	2.433355000000	0.000000000000
C	-2.433355000000	-2.433355000000	0.000000000000
C	2.433355000000	-2.433355000000	0.000000000000
C	2.433355000000	2.433355000000	0.000000000000
C	-1.120516000000	2.914455000000	0.000000000000
C	-2.914455000000	-1.120516000000	0.000000000000
C	1.120516000000	-2.914455000000	0.000000000000
C	2.914455000000	1.120516000000	0.000000000000
C	1.120516000000	2.914455000000	0.000000000000
C	-2.914455000000	1.120516000000	0.000000000000
C	-1.120516000000	-2.914455000000	0.000000000000
C	2.914455000000	-1.120516000000	0.000000000000
C	-0.704611000000	4.306855000000	0.000000000000
C	-4.306855000000	-0.704611000000	0.000000000000
C	0.704611000000	-4.306855000000	0.000000000000
C	4.306855000000	0.704611000000	0.000000000000
C	0.704611000000	4.306855000000	0.000000000000
C	-4.306855000000	0.704611000000	0.000000000000
C	-0.704611000000	-4.306855000000	0.000000000000
C	4.306855000000	-0.704611000000	0.000000000000
C	-1.411456000000	5.504556000000	0.000000000000
C	-5.504556000000	-1.411456000000	0.000000000000
C	1.411456000000	-5.504556000000	0.000000000000

C	5.504556000000	1.411456000000	0.000000000000
C	1.411456000000	5.504556000000	0.000000000000
C	-5.504556000000	1.411456000000	0.000000000000
C	-1.411456000000	-5.504556000000	0.000000000000
C	5.504556000000	-1.411456000000	0.000000000000
C	-0.699775000000	6.689999000000	0.000000000000
C	-6.689999000000	-0.699775000000	0.000000000000
C	0.699775000000	-6.689999000000	0.000000000000
C	6.689999000000	0.699775000000	0.000000000000
C	0.699775000000	6.689999000000	0.000000000000
C	-6.689999000000	0.699775000000	0.000000000000
C	-0.699775000000	-6.689999000000	0.000000000000
C	6.689999000000	-0.699775000000	0.000000000000
H	-2.495773000000	5.515502000000	0.000000000000
H	-5.515502000000	-2.495773000000	0.000000000000
H	2.495773000000	-5.515502000000	0.000000000000
H	5.515502000000	2.495773000000	0.000000000000
H	2.495773000000	5.515502000000	0.000000000000
H	-5.515502000000	2.495773000000	0.000000000000
H	-2.495773000000	-5.515502000000	0.000000000000
H	5.515502000000	-2.495773000000	0.000000000000
H	-1.230654000000	7.635111000000	0.000000000000
H	1.230654000000	7.635111000000	0.000000000000
H	-1.230654000000	-7.635111000000	0.000000000000
H	1.230654000000	-7.635111000000	0.000000000000
H	-7.635111000000	-1.230654000000	0.000000000000
H	-7.635111000000	1.230654000000	0.000000000000
H	7.635111000000	1.230654000000	0.000000000000
H	3.200309000000	-3.200309000000	0.000000000000
H	-3.200309000000	-3.200309000000	0.000000000000
H	3.200309000000	3.200309000000	0.000000000000
H	-3.200309000000	3.200309000000	0.000000000000
Cd	0.000000000000	0.000000000000	0.000000000000

Cartesian coordinates of AlClTBP optimized PBE0/def2-TZVP level of theory:

H	-7.541276000000	-1.230377000000	-0.294863000000
N	0.000000000000	2.029888000000	-0.124291000000
N	2.029888000000	0.000000000000	-0.124291000000
N	0.000000000000	-2.029888000000	-0.124291000000
N	-2.029888000000	0.000000000000	-0.124291000000
C	2.411082000000	2.411082000000	-0.146516000000
C	2.411082000000	-2.411082000000	-0.146516000000
C	-2.411082000000	-2.411082000000	-0.146516000000
C	-2.411082000000	2.411082000000	-0.146516000000
C	1.102749000000	2.836245000000	-0.148590000000
C	2.836245000000	-1.102749000000	-0.148590000000
C	-1.102749000000	-2.836245000000	-0.148590000000
C	-2.836245000000	1.102749000000	-0.148590000000
C	-1.102749000000	2.836245000000	-0.148590000000
C	2.836245000000	1.102749000000	-0.148590000000
C	1.102749000000	-2.836245000000	-0.148590000000
C	-2.836245000000	-1.102749000000	-0.148590000000

C	0.698743000000	4.219215000000	-0.191182000000
C	4.219215000000	-0.698743000000	-0.191182000000
C	-0.698743000000	-4.219215000000	-0.191182000000
C	-4.219215000000	0.698743000000	-0.191182000000
C	-0.698743000000	4.219215000000	-0.191182000000
C	4.219215000000	0.698743000000	-0.191182000000
C	0.698743000000	-4.219215000000	-0.191182000000
C	-4.219215000000	-0.698743000000	-0.191182000000
C	1.413615000000	5.413988000000	-0.230848000000
C	5.413988000000	-1.413615000000	-0.230848000000
C	-1.413615000000	-5.413988000000	-0.230848000000
C	-5.413988000000	1.413615000000	-0.230848000000
C	-1.413615000000	5.413988000000	-0.230848000000
C	5.413988000000	1.413615000000	-0.230848000000
C	1.413615000000	-5.413988000000	-0.230848000000
C	-5.413988000000	-1.413615000000	-0.230848000000
C	0.701214000000	6.595741000000	-0.266762000000
C	6.595741000000	-0.701214000000	-0.266762000000
C	-0.701214000000	-6.595741000000	-0.266762000000
C	-6.595741000000	0.701214000000	-0.266762000000
C	-0.701214000000	6.595741000000	-0.266762000000
C	6.595741000000	0.701214000000	-0.266762000000
C	0.701214000000	-6.595741000000	-0.266762000000
C	-6.595741000000	-0.701214000000	-0.266762000000
H	2.497588000000	5.424697000000	-0.231066000000
H	5.424697000000	-2.497588000000	-0.231066000000
H	-2.497588000000	-5.424697000000	-0.231066000000
H	-5.424697000000	2.497588000000	-0.231066000000
H	-2.497588000000	5.424697000000	-0.231066000000
H	5.424697000000	2.497588000000	-0.231066000000
H	2.497588000000	-5.424697000000	-0.231066000000
H	-5.424697000000	-2.497588000000	-0.231066000000
H	1.230377000000	7.541276000000	-0.294863000000
H	-1.230377000000	7.541276000000	-0.294863000000
H	1.230377000000	-7.541276000000	-0.294863000000
H	-1.230377000000	-7.541276000000	-0.294863000000
H	7.541276000000	-1.230377000000	-0.294863000000
H	7.541276000000	1.230377000000	-0.294863000000
H	-7.541276000000	1.230377000000	-0.294863000000
H	-3.176283000000	-3.176283000000	-0.163598000000
H	3.176283000000	-3.176283000000	-0.163598000000
H	-3.176283000000	3.176283000000	-0.163598000000
H	3.176283000000	3.176283000000	-0.163598000000
Ga	0.000000000000	0.000000000000	0.306726000000
Cl	0.000000000000	0.000000000000	2.502603000000

Cartesian coordinates of GaClTBP optimized PBE0/def2-TZVP level of theory:

H	-7.541276000000	-1.230377000000	-0.294863000000
N	0.000000000000	2.029888000000	-0.124291000000
N	2.029888000000	0.000000000000	-0.124291000000
N	0.000000000000	-2.029888000000	-0.124291000000
N	-2.029888000000	0.000000000000	-0.124291000000

C	2.411082000000	2.411082000000	-0.146516000000
C	2.411082000000	-2.411082000000	-0.146516000000
C	-2.411082000000	-2.411082000000	-0.146516000000
C	-2.411082000000	2.411082000000	-0.146516000000
C	1.102749000000	2.836245000000	-0.148590000000
C	2.836245000000	-1.102749000000	-0.148590000000
C	-1.102749000000	-2.836245000000	-0.148590000000
C	-2.836245000000	1.102749000000	-0.148590000000
C	-1.102749000000	2.836245000000	-0.148590000000
C	2.836245000000	1.102749000000	-0.148590000000
C	1.102749000000	-2.836245000000	-0.148590000000
C	-2.836245000000	-1.102749000000	-0.148590000000
C	0.698743000000	4.219215000000	-0.191182000000
C	4.219215000000	-0.698743000000	-0.191182000000
C	-0.698743000000	-4.219215000000	-0.191182000000
C	-4.219215000000	0.698743000000	-0.191182000000
C	-0.698743000000	4.219215000000	-0.191182000000
C	4.219215000000	0.698743000000	-0.191182000000
C	0.698743000000	-4.219215000000	-0.191182000000
C	-4.219215000000	-0.698743000000	-0.191182000000
C	1.413615000000	5.413988000000	-0.230848000000
C	5.413988000000	-1.413615000000	-0.230848000000
C	-1.413615000000	-5.413988000000	-0.230848000000
C	-5.413988000000	1.413615000000	-0.230848000000
C	-1.413615000000	5.413988000000	-0.230848000000
C	5.413988000000	1.413615000000	-0.230848000000
C	1.413615000000	-5.413988000000	-0.230848000000
C	-5.413988000000	-1.413615000000	-0.230848000000
C	0.701214000000	6.595741000000	-0.266762000000
C	6.595741000000	-0.701214000000	-0.266762000000
C	-0.701214000000	-6.595741000000	-0.266762000000
C	-6.595741000000	0.701214000000	-0.266762000000
C	-0.701214000000	6.595741000000	-0.266762000000
C	6.595741000000	0.701214000000	-0.266762000000
C	0.701214000000	-6.595741000000	-0.266762000000
C	-6.595741000000	-0.701214000000	-0.266762000000
H	2.497588000000	5.424697000000	-0.231066000000
H	5.424697000000	-2.497588000000	-0.231066000000
H	-2.497588000000	-5.424697000000	-0.231066000000
H	-5.424697000000	2.497588000000	-0.231066000000
H	-2.497588000000	5.424697000000	-0.231066000000
H	5.424697000000	2.497588000000	-0.231066000000
H	2.497588000000	-5.424697000000	-0.231066000000
H	-5.424697000000	-2.497588000000	-0.231066000000
H	1.230377000000	7.541276000000	-0.294863000000
H	-1.230377000000	7.541276000000	-0.294863000000
H	1.230377000000	-7.541276000000	-0.294863000000
H	-1.230377000000	-7.541276000000	-0.294863000000
H	7.541276000000	-1.230377000000	-0.294863000000
H	7.541276000000	1.230377000000	-0.294863000000
H	-7.541276000000	1.230377000000	-0.294863000000
H	-3.176283000000	-3.176283000000	-0.163598000000

H	3.176283000000	-3.176283000000	-0.163598000000
H	-3.176283000000	3.176283000000	-0.163598000000
H	3.176283000000	3.176283000000	-0.163598000000
Ga	0.000000000000	0.000000000000	0.306726000000
Cl	0.000000000000	0.000000000000	2.502603000000

Cartesian coordinates of InClTBP optimized PBE0/def2-TZVP level of theory:

H	-7.578144000000	-1.230307000000	-0.505118000000
N	0.000000000000	2.097605000000	-0.104371000000
N	2.097605000000	0.000000000000	-0.104371000000
N	0.000000000000	-2.097605000000	-0.104371000000
N	-2.097605000000	0.000000000000	-0.104371000000
C	2.423748000000	2.423748000000	-0.172859000000
C	2.423748000000	-2.423748000000	-0.172859000000
C	-2.423748000000	-2.423748000000	-0.172859000000
C	-2.423748000000	2.423748000000	-0.172859000000
C	1.113456000000	2.878429000000	-0.166522000000
C	2.878429000000	-1.113456000000	-0.166522000000
C	-1.113456000000	-2.878429000000	-0.166522000000
C	-2.878429000000	1.113456000000	-0.166522000000
C	-1.113456000000	2.878429000000	-0.166522000000
C	2.878429000000	1.113456000000	-0.166522000000
C	1.113456000000	-2.878429000000	-0.166522000000
C	-2.878429000000	-1.113456000000	-0.166522000000
C	0.701768000000	4.261452000000	-0.262802000000
C	4.261452000000	-0.701768000000	-0.262802000000
C	-0.701768000000	-4.261452000000	-0.262802000000
C	-4.261452000000	0.701768000000	-0.262802000000
C	-0.701768000000	4.261452000000	-0.262802000000
C	4.261452000000	0.701768000000	-0.262802000000
C	0.701768000000	-4.261452000000	-0.262802000000
C	-4.261452000000	-0.701768000000	-0.262802000000
C	1.412872000000	5.454516000000	-0.353990000000
C	5.454516000000	-1.412872000000	-0.353990000000
C	-1.412872000000	-5.454516000000	-0.353990000000
C	-5.454516000000	1.412872000000	-0.353990000000
C	-1.412872000000	5.454516000000	-0.353990000000
C	5.454516000000	1.412872000000	-0.353990000000
C	1.412872000000	-5.454516000000	-0.353990000000
C	-5.454516000000	-1.412872000000	-0.353990000000
C	0.700559000000	6.634895000000	-0.438144000000
C	6.634895000000	-0.700559000000	-0.438144000000
C	-0.700559000000	-6.634895000000	-0.438144000000
C	-6.634895000000	0.700559000000	-0.438144000000
C	-0.700559000000	6.634895000000	-0.438144000000
C	6.634895000000	0.700559000000	-0.438144000000
C	0.700559000000	-6.634895000000	-0.438144000000
C	-6.634895000000	-0.700559000000	-0.438144000000
H	2.496914000000	5.465139000000	-0.355769000000
H	5.465139000000	-2.496914000000	-0.355769000000
H	-2.496914000000	-5.465139000000	-0.355769000000
H	-5.465139000000	2.496914000000	-0.355769000000

H	-2.496914000000	5.465139000000	-0.355769000000
H	5.465139000000	2.496914000000	-0.355769000000
H	2.496914000000	-5.465139000000	-0.355769000000
H	-5.465139000000	-2.496914000000	-0.355769000000
H	1.230307000000	7.578144000000	-0.505118000000
H	-1.230307000000	7.578144000000	-0.505118000000
H	1.230307000000	-7.578144000000	-0.505118000000
H	-1.230307000000	-7.578144000000	-0.505118000000
H	7.578144000000	-1.230307000000	-0.505118000000
H	7.578144000000	1.230307000000	-0.505118000000
H	-7.578144000000	1.230307000000	-0.505118000000
H	-3.188901000000	-3.188901000000	-0.224599000000
H	3.188901000000	-3.188901000000	-0.224599000000
H	-3.188901000000	3.188901000000	-0.224599000000
H	3.188901000000	3.188901000000	-0.224599000000
In	0.000000000000	0.000000000000	0.505445000000
Cl	0.000000000000	0.000000000000	2.865866000000

Table S1. Calculated composition of the lowest excited states and corresponding oscillator strengths for H_2TBP and MTBP complexes.

State	Composition(%)	λ, nm	f
H_2TBP			
1^1B_{1u}	$2b_{3u} \rightarrow 1b_{2g}^*$ (31)	578	0.11
	$3a_u \rightarrow 1b_{1g}^*$ (69)		
1^1B_{2u}	$2b_{3u} \rightarrow 1b_{1g}^*$ (19)	568	0.23
	$3a_u \rightarrow 1b_{2g}^*$ (81)		
2^1B_{1u}	$2b_{3u} \rightarrow 1b_{2g}^*$ (8)	387	1.24
	$2b_{3u} \rightarrow 1b_{2g}^*$ (59)		
	$3a_u \rightarrow 1b_{1g}^*$ (28)		
2^1B_{2u}	$2b_{3u} \rightarrow 1b_{1g}^*$ (74)	372	1.10
	$3a_u \rightarrow 1b_{2g}^*$ (18)		
3^1B_{1u}	$2b_{3u} \rightarrow 1b_{2g}^*$ (88)	332	0.35
	$2b_{3u} \rightarrow 1b_{2g}^*$ (6)		
3^1B_{2u}	$3a_u \rightarrow 2b_{2g}^*$ (93)	331	0.15
4^1B_{2u}	$2b_{3u} \rightarrow 1b_{1g}^*$ (95)	314	0.18
6^1B_{1u}	$1b_{3u} \rightarrow 1b_{2g}^*$ (73)	281	0.22
	$2a_u \rightarrow 1b_{1g}^*$ (24)		
8^1B_{2u}	$1a_u \rightarrow 1b_{2g}^*$ (90)	257	0.11
10^1B_{1u}	$2b_{3u} \rightarrow 2b_{2g}^*$ (5)	230	0.08
	$2b_{1g} \rightarrow 1a_u^*$ (87)		
12^1B_{2u}	$1b_{1g} \rightarrow 1b_{3u}^*$ (20)	217	0.49
	$2b_{2g} \rightarrow 1a_u^*$ (5)		
	$2b_{1g} \rightarrow 2b_{3u}^*$ (15)		
	$2b_{3u} \rightarrow 3b_{1g}^*$ (54)		
11^1B_{1u}	$2b_{2g} \rightarrow 1b_{3u}^*$ (93)	216	0.06
12^1B_{1u}	$1b_{2g} \rightarrow 1b_{3u}^*$ (7)	211	0.26
	$1b_{1g} \rightarrow 1a_u^*$ (17)		
	$2b_{3u} \rightarrow 2b_{2g}^*$ (50)		
	$3a_u \rightarrow 3b_{1g}^*$ (5)		
13^1B_{1u}	$1b_{3u} \rightarrow 2b_{2g}^*$ (6)	209	0.38
	$1b_{2g} \rightarrow 1b_{3u}^*$ (11)		
	$1b_{1g} \rightarrow 1a_u^*$ (22)		
	$2b_{3u} \rightarrow 2b_{2g}^*$ (43)		
	$2b_{1g} \rightarrow 1a_u^*$ (6)		
15^1B_{2u}	$2a_u \rightarrow 2b_{2g}^*$ (11)	208	0.19
	$2b_{2g} \rightarrow 1a_u^*$ (23)		
	$2b_{3u} \rightarrow 2b_{1g}^*$ (36)		
	$2b_{3u} \rightarrow 3b_{1g}^*$ (12)		
	$3a_u \rightarrow 3b_{2g}^*$ (5)		
16^1B_{2u}	$1b_{2g} \rightarrow 1a_u^*$ (19)	203	0.22
	$2a_u \rightarrow 2b_{2g}^*$ (49)		
	$1b_{1g} \rightarrow 1b_{3u}^*$ (8)		
	$1b_{1g} \rightarrow 2b_{3u}^*$ (6)		
14^1B_{1u}	$1b_{1g} \rightarrow 1a_u^*$ (12)	201	0.17
	$2b_{2g} \rightarrow 2b_{3u}^*$ (5)		
	$2b_{3u} \rightarrow 143$ (79)		
18^1B_{2u}	$1b_{2g} \rightarrow 1a_u^*$ (62)	201	0.12

State	Composition(%)	λ, nm	f
	$2a_u \rightarrow 2b^*_{2g}$ (18)		
	$1b_{1g} \rightarrow 2b^*_{3u}$ (14)		
ZnTBP			
1^1E_u	$2a_{2u} \rightarrow 1e^*_g$ (21)	563	0.19
	$2a_{1u} \rightarrow 1e^*_g$ (79)		
2^1E_u	$2a_{2u} \rightarrow 1e^*_g$ (73)	372	1.17
	$2a_{1u} \rightarrow 1e^*_g$ (20)		
3^1E_u	$2a_{1u} \rightarrow 2e^*_g$ (94)	326	0.13
6^1E_u	$1a_{2u} \rightarrow 1e^*_g$ (23)	287	0.14
	$1b_{1u} \rightarrow 1e^*_g$ (68)		
	$1b_{2u} \rightarrow 1e^*_g$ (6)		
8^1E_u	$1a_{1u} \rightarrow 1e^*_g$ (87)	252	0.08
10^1E_u	$1a_{1u} \rightarrow 1e^*_g$ (5)	225	0.12
	$1e_g \rightarrow 1b^*_{1u}$ (5)		
	$2e_g \rightarrow 1b^*_{1u}$ (24)		
	$2e_g \rightarrow 1a^*_{2u}$ (30)		
	$2a_{1u} \rightarrow 3e^*_g$ (23)		
12^1E_u	$1e_g \rightarrow 1b^*_{2u}$ (5)	221	0.14
	$2e_g \rightarrow 1b^*_{1u}$ (10)		
	$2e_g \rightarrow 1b^*_{2u}$ (55)		
	$2e_g \rightarrow 1a^*_{2u}$ (22)		
13^1E_u	$1e_g \rightarrow 1b^*_{1u}$ (19)	216	0.27
	$1e_g \rightarrow 1b^*_{2u}$ (27)		
	$1b_{2u} \rightarrow 2e^*_g$ (10)		
	$2e_g \rightarrow 1b^*_{1u}$ (15)		
	$2e_g \rightarrow 1b^*_{2u}$ (6)		
	$2e_g \rightarrow 1a^*_{2u}$ (10)		
	$2a_{2u} \rightarrow 3e^*_g$ (5)		
14^1E_u	$1e_g \rightarrow 1b^*_{2u}$ (12)	212	0.45
	$1e_g \rightarrow 1a^*_{2u}$ (62)		
	$2e_g \rightarrow 1b^*_{1u}$ (7)		
	$2a_{2u} \rightarrow 3e^*_g$ (12)		
17^1E_u	$1a_{2u} \rightarrow 2e^*_g$ (6)	205	0.15
	$1e_g \rightarrow 1b^*_{1u}$ (45)		
	$1e_g \rightarrow 1b^*_{2u}$ (12)		
	$1b_{2u} \rightarrow 2e^*_g$ (14)		
	$2a_{2u} \rightarrow 3e^*_g$ (15)		
CdTBP			
1^1E_u	$2a_{2u} \rightarrow 1e^*_g$ (23)	560	0.18
	$2a_{1u} \rightarrow 1e^*_g$ (77)		
2^1 E_u	$2a_{2u} \rightarrow 1e^*_g$ (71)	376	1.21
	$2a_{1u} \rightarrow 1e^*_g$ (22)		
3^1E_u	$1b_{2u} \rightarrow 1e^*_g$ (18)	320	0.08
	$2a_{1u} \rightarrow 2e^*_g$ (78)		
5^1E_u	$1b_{1u} \rightarrow 1e^*_g$ (5)	295	0.08
	$1a_{2u} \rightarrow 1e^*_g$ (86)		
	$1b_{2u} \rightarrow 1e^*_g$ (5)		
6^1E_u	$1b_{1u} \rightarrow 1e^*_g$ (88)	290	0.09
	$1a_{2u} \rightarrow 1e^*_g$ (5)		

State	Composition(%)	λ, nm	f
8^1E_u	$1a_{1u} \rightarrow 1e_g^*$ (87)	253	0.09
11^1E_u	$1e_g \rightarrow 1b_{1u}^*$ (13)	224	0.13
	$2e_g \rightarrow 1b_{2u}^*$ (12)		
	$2e_g \rightarrow 1a_{2u}^*$ (52)		
	$2e_g \rightarrow 1b_{1u}^*$ (10)		
	$2a_{1u} \rightarrow 3e_g^*$ (5)		
12^1E_u	$2e_g \rightarrow 1b_{2u}^*$ (71)	220	0.26
	$2e_g \rightarrow 1a_{2u}^*$ (11)		
	$2e_g \rightarrow 1b_{1u}^*$ (5)		
13^1E_u	$1e_g \rightarrow 1b_{1u}^*$ (27)	217	0.14
	$1e_g \rightarrow 1b_{2u}^*$ (21)		
	$1b_{2u} \rightarrow 2e_g^*$ (6)		
	$2e_g \rightarrow 1b_{2u}^*$ (6)		
	$2e_g \rightarrow 1a_{2u}^*$ (23)		
	$2e_g \rightarrow 1b_{1u}^*$ (6)		
14^1E_u	$1e_g \rightarrow 1b_{1u}^*$ (10)	213	0.45
	$1e_g \rightarrow 1a_{2u}^*$ (25)		
	$2a_{2u} \rightarrow 3e_g^*$ (58)		
16^1E_u	$1e_g \rightarrow 1b_{1u}^*$ (10)	208	0.06
	$1e_g \rightarrow 1b_{2u}^*$ (40)		
	$1e_g \rightarrow 1a_{2u}^*$ (10)		
	$1b_{2u} \rightarrow 2e_g^*$ (26)		
	$2a_{2u} \rightarrow 3e_g^*$ (7)		
17^1E_u	$1a_{2u} \rightarrow 2e_g^*$ (5)	206	0.12
	$1e_g \rightarrow 1b_{1u}^*$ (26)		
	$1b_{2u} \rightarrow 2e_g^*$ (52)		
	$2a_{2u} \rightarrow 3e_g^*$ (10)		
AlClTBP			
1^1E	$2a_1 \rightarrow 1e^*$ (20)	580	0.18
	$2a_2 \rightarrow 1e^*$ (80)		
2^1E	$2a_1 \rightarrow 1e^*$ (73)	382	1.01
	$2a_2 \rightarrow 1e^*$ (19)		
3^1E	$2a_2 \rightarrow 2e^*$ (93)	329	0.14
5^1E	$1b_2 \rightarrow 1e^*$ (17)	297	0.08
	$1a_1 \rightarrow 1e^*$ (74)		
	$1b_1 \rightarrow 1e^*$ (5)		
6^1E	$1b_2 \rightarrow 1e^*$ (75)	291	0.19
	$1a_1 \rightarrow 1e^*$ (14)		
	$1b_1 \rightarrow 1e^*$ (8)		
8^1E	$1a_2 \rightarrow 1e^*$ (90)	254	0.06
11^1E	$3e \rightarrow 1b_2^*$ (17)	225	0.17
	$3e \rightarrow 1b_1^*$ (59)		
	$2a_2 \rightarrow 3e^*$ (15)		
13^1E	$3e \rightarrow 1b_2^*$ (25)	219	0.11
	$3e \rightarrow 1b_1^*$ (8)		
	$3e \rightarrow 1a_1^*$ (49)		
17^1E	$2e \rightarrow 1b_1^*$ (13)	212	0.19
	$2e \rightarrow 1a_1^*$ (23)		
	$2e \rightarrow 1b_2^*$ (15)		
	$1b_1 \rightarrow 2e^*$ (14)		
	$3e \rightarrow 1b_2^*$ (9)		
	$3e \rightarrow 1a_1^*$ (14)		

State	Composition(%)	λ, nm	f
18[1]E	$2e \rightarrow 1b_1^*$ (6)	211	0.22
	$2e \rightarrow 1a_1^*$ (47)		
	$2e \rightarrow 1b_2^*$ (11)		
	$1b_1 \rightarrow 2e^*$ (11)		
	$2a_1 \rightarrow 3e^*$ (9)		
19[1]E	$2e \rightarrow 1a_1^*$ (5)	209	0.14
	$2e \rightarrow 1b_2^*$ (12)		
	$1b_1 \rightarrow 2e^*$ (16)		
	$2a_1 \rightarrow 3e^*$ (61)		
21[1]E	$1a_1 \rightarrow 2e^*$ (55)	203	0.11
	$2e \rightarrow 1b_2^*$ (18)		
	$3e \rightarrow 1b_2^*$ (7)		
	$2a_1 \rightarrow 3e^*$ (7)		
23[1]E	$1e \rightarrow 1b_1^*$ (6)	201	0.24
	$1b_2 \rightarrow 2e^*$ (80)		
GaClTBP			
1[1]E	$3a_1 \rightarrow 1e^*$ (21)	577	0.17
	$2a_2 \rightarrow 1e^*$ (79)		
2[1]E	$3a_1 \rightarrow 1e^*$ (73)	385	0.99
	$2a_2 \rightarrow 1e^*$ (20)		
3[1]E	$2b_1 \rightarrow 1e^*$ (5)	326	0.10
	$2a_2 \rightarrow 2e^*$ (93)		
5[1]E	$1b_2 \rightarrow 1e^*$ (10)	299	0.14
	$2a_1 \rightarrow 1e^*$ (77)		
	$2b_1 \rightarrow 1e^*$ (5)		
6[1]E	$1b_2 \rightarrow 1e^*$ (83)	292	0.17
	$2a_1 \rightarrow 1e^*$ (6)		
	$2b_1 \rightarrow 1e^*$ (7)		
9[1]E	$1a_2 \rightarrow 1e^*$ (90)	255	0.06
11[1]E	$1a_1 \rightarrow 1e^*$ (7)	224	0.17
	$3e \rightarrow 1b_1^*$ (7)		
	$4e \rightarrow 1b_2^*$ (34)		
	$4e \rightarrow 1b_1^*$ (8)		
	$2a_2 \rightarrow 3e^*$ (22)		
14[1]E	$1a_1 \rightarrow 1e^*$ (5)	218	0.23
	$3e \rightarrow 1b_2^*$ (8)		
	$4e \rightarrow 1b_2^*$ (34)		
	$4e \rightarrow 1b_1^*$ (9)		
	$4e \rightarrow 1a_1^*$ (34)		
17[1]E	$2e \rightarrow 1b_1^*$ (5)	211	0.24
	$3e \rightarrow 1b_2^*$ (11)		
	$3e \rightarrow 1b_1^*$ (12)		
	$3e \rightarrow 1a_1^*$ (19)		
	$2b_1 \rightarrow 2e^*$ (11)		
	$4e \rightarrow 1b_2^*$ (7)		
	$4e \rightarrow 1a_1^*$ (19)		
	$3a_1 \rightarrow 3e^*$ (8)		
18[1]E	$3e \rightarrow 1b_2^*$ (23)	211	0.25
	$3e \rightarrow 1b_1^*$ (5)		
	$3e \rightarrow 1a_1^*$ (9)		
	$3a_1 \rightarrow 3e^*$ (54)		
23[1]E	$2e \rightarrow 1b_2^*$ (13)	200	0.19
	$1b_2 \rightarrow 2e^*$ (75)		
InClTBP			

State	Composition(%)	λ, nm	f
1^1E	$2a_1 \rightarrow 1e^*$ (22)	577	0.17
	$2a_2 \rightarrow 1e^*$ (77)		
2^1E	$2a_1 \rightarrow 1e^*$ (72)	388	1.03
	$2a_2 \rightarrow 1e^*$ (22)		
3^1E	$1b_1 \rightarrow 1e^*$ (23)	322	0.07
	$2a_2 \rightarrow 2e^*$ (73)		
5^1E	$1a_1 \rightarrow 1e^*$ (79)	303	0.16
	$1b_1 \rightarrow 1e^*$ (12)		
6^1E	$1b_2 \rightarrow 1e^*$ (87)	295	0.15
	$1b_1 \rightarrow 1e^*$ (6)		
9^1E	$1a_2 \rightarrow 1e^*$ (90)	256	0.06
15^1E	$2e \rightarrow 1b_2^*$ (10)	217	0.28
	$3e \rightarrow 1b_1^*$ (25)		
	$3e \rightarrow 1a_1^*$ (34)		
	$3e \rightarrow 1b_2^*$ (14)		
	$2a_2 \rightarrow 4e^*$ (5)		
16^1E	$2e \rightarrow 1b_1^*$ (43)	214	0.14
	$2e \rightarrow 1b_2^*$ (7)		
	$3e \rightarrow 1a_1^*$ (35)		
	$3e \rightarrow 1b_2^*$ (6)		
17^1E	$2e \rightarrow 1a_1^*$ (10)	211	0.27
	$2e \rightarrow 1a_1^*$ (10)		
	$2a_1 \rightarrow 3e^*$(71)		
18^1E	$2e \rightarrow 1b_1^*$ (26)	210	0.07
	$2e \rightarrow 1a_1^*$ (22)		
	$2e \rightarrow 1b_2^*$ (16)		
	$1b_1 \rightarrow 2e^*$ (8)		
	$3e \rightarrow 1a_1^*$ (15)		
19^1E	$1e \rightarrow 1b_1^*$ (11)	208	0.15
	$1e \rightarrow 1a_1^*$ (7)		
	$2e \rightarrow 1b_1^*$ (7)		
	$2e \rightarrow 1a_1^*$ (35)		
	$1b_1 \rightarrow 2e^*$ (15)		
	$3e \rightarrow 1b_2^*$ (8)		
	$2a_1 \rightarrow 3e^*$ (6)		
20^1E	$1a_1 \rightarrow 2e^*$ (12)	207	0.10
	$2e \rightarrow 1b_2^*$ (24)		
	$1b_1 \rightarrow 2e^*$ (48)		
	$2a_1 \rightarrow 3e^*$ (6)		
21^1E	$1e \rightarrow 1b_1^*$ (75)	204	0.06
	$2e \rightarrow 1b_2^*$ (6)		
